# The interplay between migration and self-identity: a structured review using TCCM and bibliometric analysis

**DOI:** 10.3389/fpsyg.2025.1563508

**Published:** 2025-07-08

**Authors:** Harshita Barua, Nidhi Maheshwari

**Affiliations:** ^1^Centre of Excellence for the Science of Happiness, Delhi Technological University (DTU), Main Campus, Delhi, India; ^2^University School of Management and Entrepreneurship (USME), Delhi Technological University (DTU), East Delhi Campus, Delhi, India

**Keywords:** migration, self-identity, acculturation, bibliometric review, co-citation, TCCM, thematic mapping, cultural adaptation

## Abstract

**Introduction:**

This study explores the intricate relationship between migration and self-identity, emphasizing how the migratory process extends beyond geographic movement to impact individuals' cultural, social, and psychological landscapes. As migrants adapt to new environments, shifts in language, traditions, and social norms may challenge their sense of belonging and self-concept, potentially resulting in identity conflicts and reduced confidence-particularly in workplace settings.

**Methods:**

A bibliometric review was conducted to systematically analyze the literature on migration and self-identity. Data were sourced from the Web of Science database. Analytical tools such as VOSviewer and Biblioshiny were employed to map co-citation patterns, thematic clusters, and emerging trends. Additionally, the Theory-Context-Characteristics-Methodology (TCCM) framework guided the synthesis of research gaps and future directions.

**Results:**

The analysis identified dominant research themes such as acculturation, identity negotiation, and sociocultural adaptation challenges. Most studies focus on Western host societies and adult migrant populations. However, there is a growing yet underexplored body of work on second-generation migrants and identity development in non-Western contexts.

**Discussion:**

Findings suggest a pressing need for more inclusive research across diverse cultural settings. Practically, organizations should recognize the impact of migration on self-identity and implement culturally sensitive policies to support migrant integration. Managerial implications include promoting inclusive workplace cultures through diversity training and mental health support programs.

## 1 Introduction

Migration is an age-old phenomenon that has existed since the beginning of human civilization, playing a crucial role in shaping populations and transforming landscapes. It is a multifaceted process encompassing the movement of people or groups from one location to another, generally for reasons like political turmoil, economic prospects, better living conditions, overcoming poverty, or employment opportunities (Virupakshas et al., 2014; Castelli, 2018; Choy et al., 2021). Migration is more than just relocating, it involves profound cultural, social, and psychological adjustments (Brance et al., 2024).

As individuals transition into new environments, they often face uncertainty, requiring them to adapt and restructure their social networks. Thus, migration can become a catalyst for rethinking and renegotiating one's identity (La Barbera, 2015). It is the interplay between how we see ourselves, i.e, self-representation, and how others categorize us, i.e, social categorization (Simon, 2004; Deaux, 1993). This identity is intimately tied to cultural, social, and personal experiences. Migrant's sense of self can be profoundly affected by their new circumstances, leading to psychological and emotional distress (O'Brien et al., 2000; Bak-Klimek et al., 2018). Social and cultural dislocation can cause a loss of familiar support networks, while economic challenges may exacerbate feelings of instability. Regardless of positive or negative associations with life changes, significant life transitions are often associated with migration, requiring people to adjust and reorient themselves in the new environment, which can negatively influence people's wellbeing (Carleton et al., 2007).

Psychologically, the migrants may grapple with identity crisis, heightened stress, self-esteem issues, and a sense of alienation as they navigate new cultural landscapes (Altinyelken, 2009; Krishnaveni, 2010; Virupakshas et al., 2014). On a sociological level, the clash between their native cultural norms and the host society's expectations can lead to issues in social integration and community cohesion. Economically, the struggle to adapt to a new job market and socioeconomic environment can exacerbate insecurity and undermine self-esteem. The process of migration often leads to a transformation in self-identity, which can have long-term effects on both individual wellbeing and social integration. When migrants experience cultural shifts, social isolation, or identity conflicts, their sense of self may feel disrupted. Thus, in the age of migration, identity is not an inherited, ascribed, nor achieved status that matters, but the status that one “maintains” in any given place and time in the process of fitting oneself into a community of “strangers” (Horenczyk and Tatar, 2012; Castelli, 2018).

The central concept for understanding the psychological and social challenges of migration is identity. At its core, identity refers to how individuals understand and define themselves—how they answer the question, “*Who am I?”* Psychologically, identity has often been viewed as a relatively stable set of meanings, roles, and affiliations through which a person organizes their sense of self. Deaux (1993), for instance, defines identity as comprising the social categories individuals claim membership in (such as gender, ethnicity, religion, or profession) and the subjective meanings they attach to those categories. In this classical view, identity provides a sense of coherence and continuity, shaping one's goals, values, and relationships across time.

More recent approaches have taken a different path by highlighting the deeper and more complex ways identity changes during migration. Acculturation is now seen not just as a personal or neutral psychological process but as something shaped by power dynamics and global systems that influence how migrants are seen and treated in their new societies (Bhatia and Ram, 2009). Older models also often assume culture is something static and clearly defined, when in reality, culture is always changing and can mean different things in different situations. Chirkov (2009) points out that these models should be updated to reflect the real-life diversity of migrant experiences and to move beyond the narrow, Western-centered views of identity.

Similarly, identity in the migration context is increasingly understood as a deeply situated process, shaped not only by interpersonal experiences but also by broader geopolitical forces and collective histories. Migrants are not merely adapting to new environments but are simultaneously asserting agency, negotiating belonging, and resisting marginalization within systems that may deny or devalue their cultural identities.

This complex interplay between identity and migration can produce significant psychological strain. Migrants often face heightened levels of stress, alienation, and identity confusion, particularly when their self-concept is challenged by conflicting cultural expectations (Altinyelken, 2009; Krishnaveni, 2010; Thukral et al., 2020). Socially, the disjunction between familiar norms and the demands of the host society can hinder integration and lead to experiences of exclusion. Economic instability, language barriers, and systemic discrimination may further undermine self-esteem and exacerbate the challenges of forming a coherent and resilient sense of self.

Consequently, identity transformation in migratory contexts should be conceptualized not as a linear path toward integration but as a multidimensional and contested journey involving both loss and creative adaptation. It is influenced by an interplay of personal agency, structural constraints, and cultural negotiation. Supporting migrants through this process requires not only tangible resources and institutional support but also culturally responsive frameworks that validate the richness and diversity of their identities (Foo et al., 2018).

Despite increasing scholarly attention to these dynamics, much of the existing literature still lacks systematic integration of these critical approaches. The current study seeks to address this gap by conducting a bibliometric analysis of the literature on migration and self-identity. Through mapping key research patterns, theoretical frameworks, and methodological trends, the study aims to offer a comprehensive overview of the field and highlight areas for future inquiry that better reflect the evolving and complex realities of migrant identity formation.

### 1.1 RQ1: what are the prevailing trends and patterns in the past literature on the interplay between migration and self-identity?

The first research question is designed to systematically explore the historical development of this body of literature over time as revealed through a bibliometric analysis, including co-authorship, country wise and year-wise citation trends and applying TCCM framework to address the proposed research questions and provide deeper insights into the theoretical foundations, contexts, characteristics, and methodologies present in the literature on the interplay between self-identity and migration.

### 1.2 RQ2: what are the dominant research topics in the study of migration's impact on self-identity?

This second research question aims to pinpoint key research areas and topics where significant work has been conducted, using keyword co-occurrence and most relevant words to offer valuable insights into the core themes and emerging trends in the field.

### 1.3 RQ3: what are the primary themes or areas in the study of migration's impact on self-identity, as identified through thematic mapping of current literature?

Finally, the third research question seeks to identify potential future research avenues that could address current gaps in the literature and suggest new perspectives on how to better nurture the self-identity of migrants through thematic mapping.

## 2 Research methodology

A bibliometric review was conducted as the research methodology for this study. According to Cobo et al. (2011), bibliometric analysis offers objective criteria that surpass traditional bibliographic review methods by enabling the assessment of research development within a particular field. It serves as a valuable tool for measuring the quality and productivity of knowledge production. This approach is essential for evaluating the research contributions of various actors, including countries, universities, research centers, research groups, journals, and individual researchers.

### 2.1 Selecting a database for data extraction

The first step in conducting a bibliometric analysis is selecting the appropriate database to retrieve relevant documents. For this study, we utilized the Web of Science Database, which is known for its extensive coverage. It features more than 21,000 peer-reviewed and high-quality scholarly journals published worldwide (including Open Access journals), over 2,05,000 conference proceedings, and over 1,04,000 editorially selected books in more than 45 languages, spanning the areas of sciences, social sciences, and arts and humanities. It includes prestigious indexes like the Science Citation Index Expanded (SCIE), Social Sciences Citation Index (SSCI), and Arts and Humanities Citation Index (A&HCI), among others. The search can be performed using terms in titles, abstracts, journal names, author names, or affiliations.

**Figure 1:**
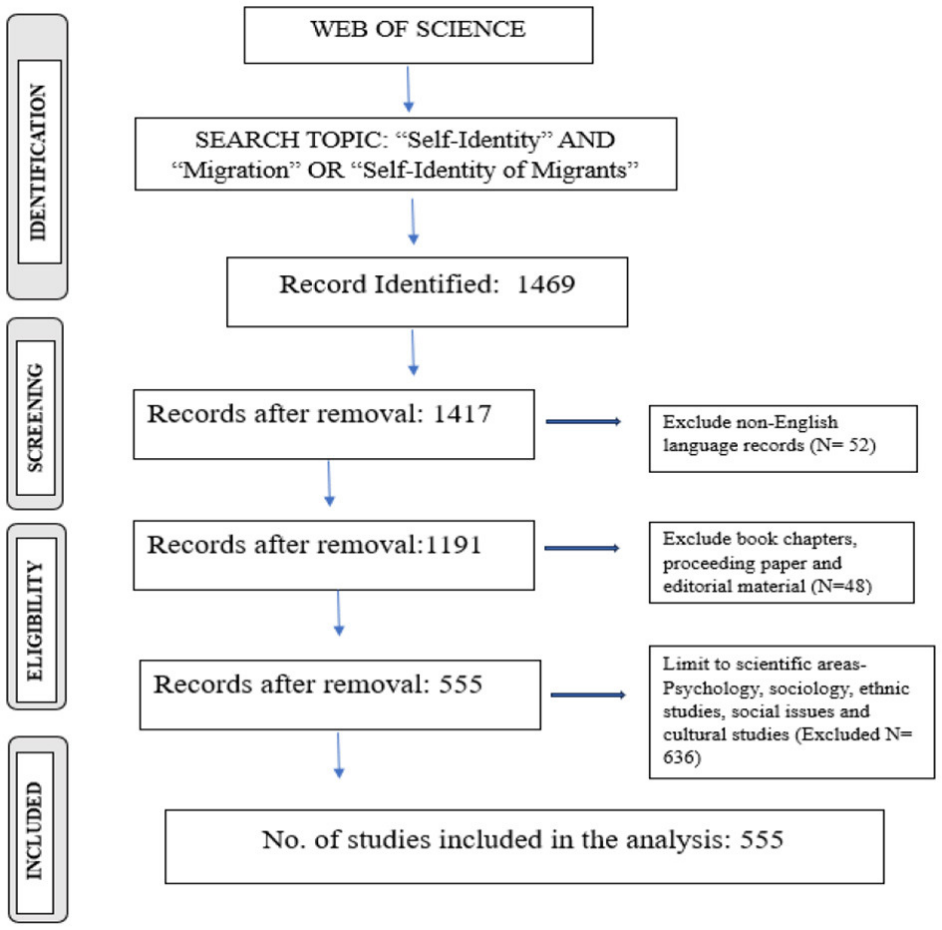
Schematic depiction of the article selection process. Source: Author's own work.

We followed the framework developed by Moher et al. (2009) and used the PRISMA (Preferred Reporting Items for Systematic Reviews and Meta-Analyses) flow diagram to guide the systematic selection and categorization of articles. The PRISMA diagram (illustrated in Figure 1) was employed to visually represent the screening process, showing the records evaluated and the exclusion criteria applied to determine article eligibility.

### 2.2 Search strategy

A well-structured search query is essential for retrieving relevant documents while minimizing irrelevant results. To explore the “impact of migration on self-identity,” we reviewed systematic reviews and bibliometric analyses to refine our search. Key terms like “self-identity,” “identity,” “migrants,” and “migration” were used with Boolean operators (OR, AND) to focus on identity crises in migration while filtering out unrelated topics. The search targeted titles, abstracts, and keywords to ensure accuracy and relevance.

To conduct a comprehensive bibliometric review for this research, a systematic methodology was employed. According to Cobo et al. (2011), bibliometric analysis offers objective criteria, unlike traditional bibliographic reviews, which help in assessing research development and measuring the quality and productivity of knowledge production. This approach is particularly useful for evaluating research contributions from various entities, including countries, universities, research centers, journals, and individual researchers. In the present study, the search was conducted with specific inclusion and exclusion criteria: (1) a time frame spanning from 2004 to 2024 was utilized to capture relevant publications on self-identity and migration; (2) book chapters and editorial material were excluded, focusing instead on articles and reviews for their currency and relevance; (3) the search was limited to English-language publications, as English is the predominant language in scientific discourse and widely used in reviews (López-Fernández et al., 2016); and (4) the search was restricted to specific fields: social Sciences, Psychology (multidisciplinary), Sociology, Ethnic Studies, and Arts and Humanities, as categorized by the Web of Science. The initial search identified 1,469 records. After removing non-English language documents (*N* = 52), the total was reduced to 1,417 records. Further exclusion of book chapters and editorial material (*N* = 48) brought the count down to 1,191. Limiting the search to the specified scientific areas (Social Sciences, Psychology, Demography, Sociology, Ethnic Studies, Management, Cultural Studies) led to the exclusion of 636 records, resulting in a final dataset of 555 articles.

Consistent with Aria and Cuccurullo's (2017) methodology, a bibliometric analysis was conducted using the R Studio 4.0.4 software tool with Bibliometrix version 3.0.4. The Bibliometrix package in R offers a robust and adaptable tool for handling and visualizing bibliometric data (Aria and Cuccurullo, 2017). Vosviewer was also employed for some steps. VOSviewer is capable of mapping various types of bibliometric analyses, supports several key bibliographic databases, disregards the time dimension, and is limited to analyzing small to medium-sized datasets

## 3 Results

### 3.1 RQ1: what are the prevailing trends and patterns in the past literature on the interplay between migration and self-identity?

The first research question aims to systematically examine the historical progression of this body of literature using a bibliometric analysis. This approach will involve applying the TCCM framework to address the research questions and offer deeper insights into the theoretical foundations, contexts, characteristics, and methodologies used in studies related to the relationship between self-identity and migration.

#### 3.1.1 TCCM

**Figure 2:**
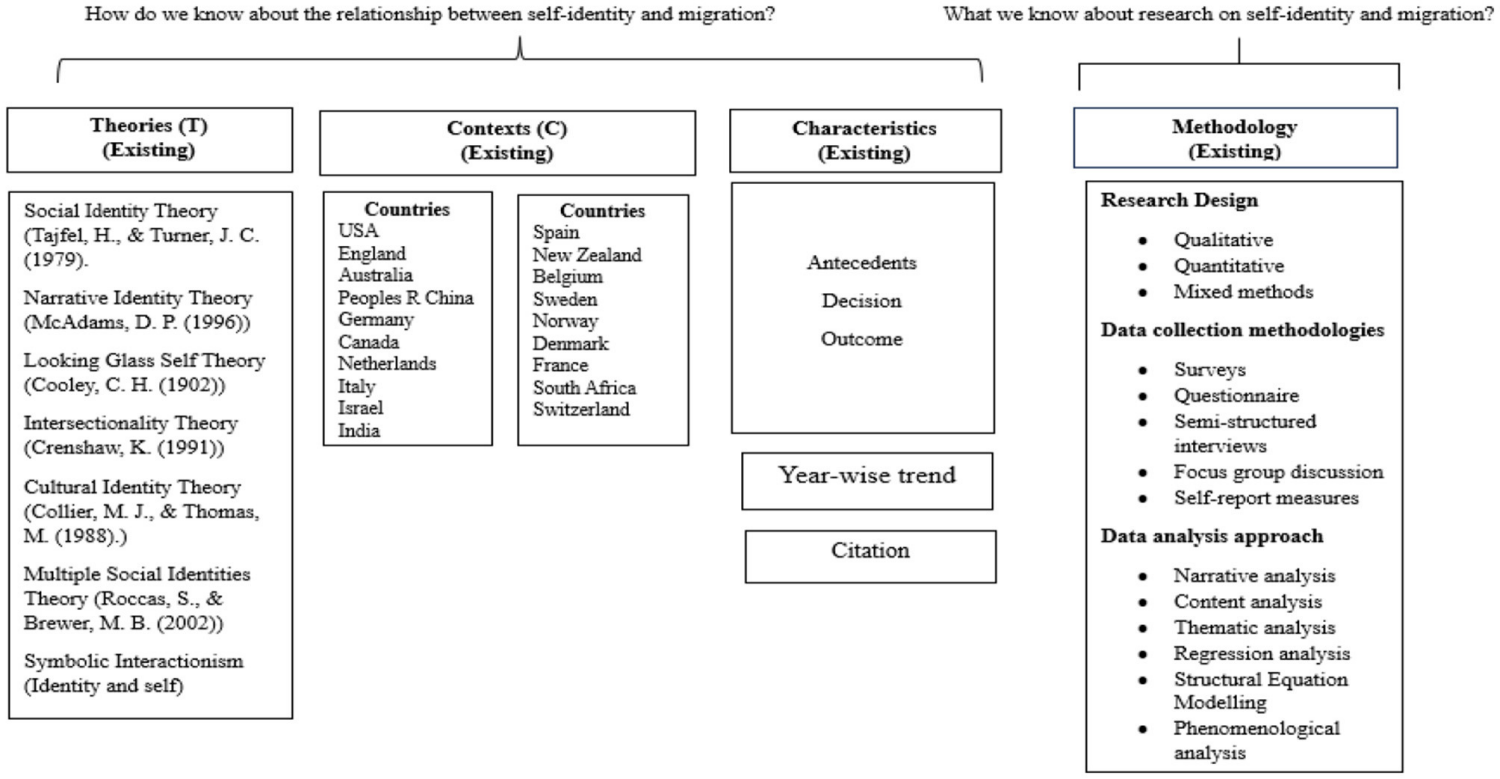
The TCCM framework (Paul and Rosado-Serrano, 2019) was adopted for this study. Source: Author's own work.

TCCM Analysis helps in bridging the gaps identified in the previous studies and offers pathways for further scope (Rajan and Dhir, 2020). Figure 2 shows how the application of the TCCM framework helps answer the proposed research questions.

**i. Theories (T)**

Social Identity Theory (Tajfel and Turner, 1979) remains a cornerstone for examining identity formation and group behavior. According to this theory, migrants categorize themselves and others into groups (e.g., based on nationality, religion, gender, etc.), and this group membership is a critical part of their self-concept (Henríquez et al., 2021; Brance et al., 2024). Once categorized, individuals adopt the identity of the group to which they belong. This identification leads to the internalization of group norms and values, which influence behavior and self-perception (Hamidou-Schmidt and Mayer, 2021). Similarly, Looking Glass Self Theory (Cooley, 1902) suggests that individuals' self-views are shaped by societal views of their group. Looking Glass Self Theory (Cooley, 1902) complements SIT by emphasizing how migrants' self-perceptions are influenced by how they believe others perceive them. A migrant might begin to see themselves through the “mirror” of public attitudes, whether those views are welcoming or prejudiced. For instance, if migrants sense that society views their ethnic group as inferior or problematic, this can shape their internal self-image negatively, unless they have strong personal or community-based buffers. However, Crocker et al. (1994) found that ethnic groups differ in how much their personal (private) and societal (public) views align. Some migrants may have a strong private sense of pride and identity, even when public perception is negative, helping them resist assimilation pressures or stigma.

Intersectionality Theory (Crenshaw, 1991) and Cultural Identity Theory (Collier and Thomas, 1988) highlight how various identity factors and cultural contexts shape migrant experiences. For instance, a female migrant of color who is undocumented may face multiple layers of discrimination—based on her gender (sexism), race (racism), and immigration status (xenophobia or legal exclusion). Her experience cannot be fully understood by looking at any one of these factors in isolation. By contrast, a male, middle-class migrant with legal status may have greater access to resources, social mobility, and safety, even if he faces some cultural adjustment challenges. Intersectionality theory encourages us to look beyond generalizations about “migrants” and pay attention to how intersecting identities shape different migration experiences and outcomes, including identity formation, inclusion, and access to rights.

Multiple Social Identities Theory (Roccas and Brewer, 2002) further explores how migrants navigate and balance their different identities. This dual identification can be enriching and allow for broader belonging. However, it can also create internal conflict if the values or expectations of the groups clash. For example, a young migrant might feel torn between traditional family expectations and more liberal norms in the host country. Meanwhile, Narrative Identity Theory (McAdams, 1996) focuses on how social interactions and personal stories influence the development of identity. Migrants often reflect on their migration journey and incorporate it into a personal narrative that explains who they are, where they came from, and what they hope for the future. These narratives give meaning to their experiences, whether of trauma, resilience, adaptation, or success.

Despite these advancements, there is still a need for deeper exploration into the complexities of identity responsiveness.

**ii. Characteristics**

**Annual trends in scientific production**

**Figure 3:**
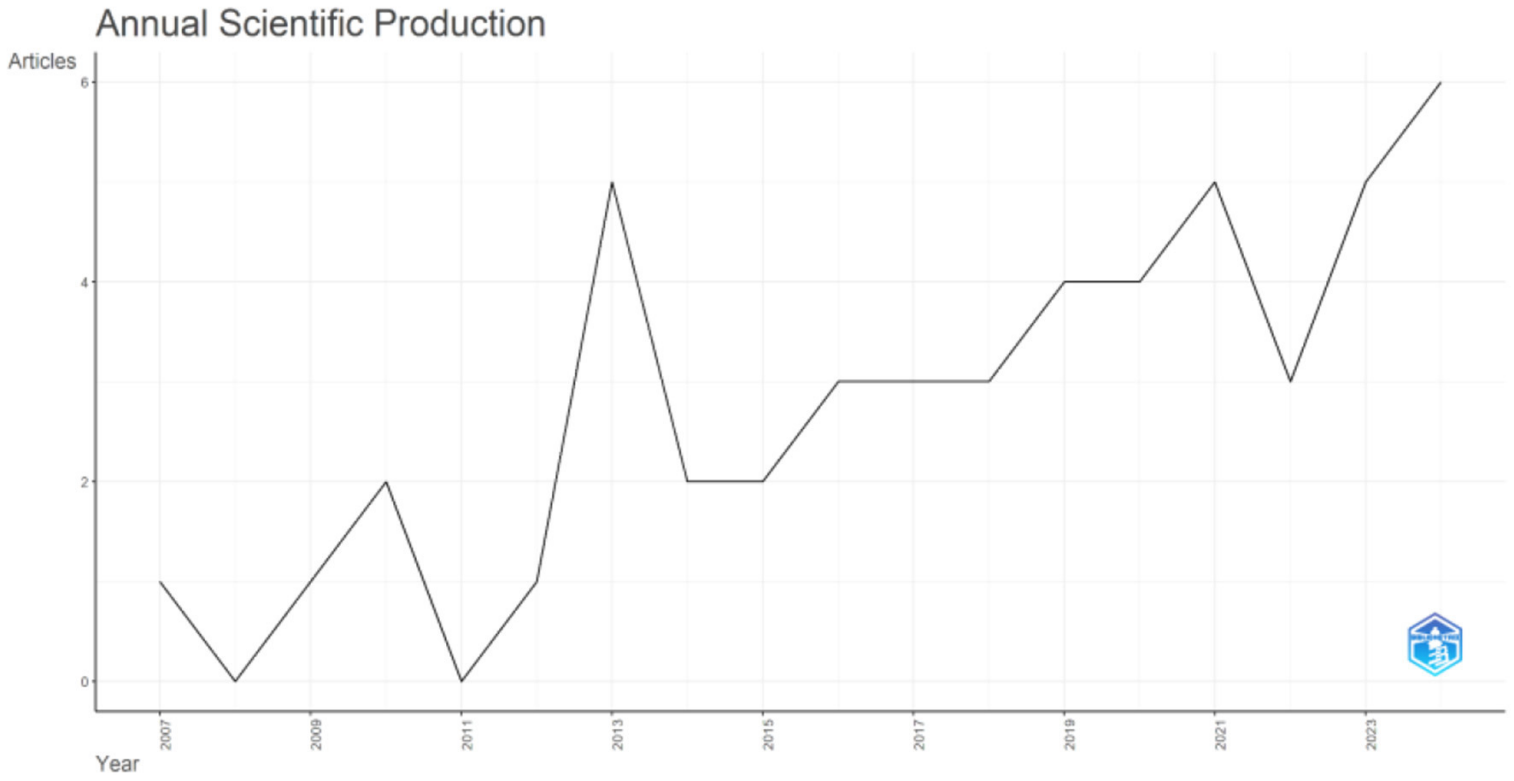
Annual scientific production of articles from 2007–2024.

The annual scientific production from 2007 to 2023 shows fluctuating research activity, with a slow start from 2007 to 2012 as depicted in Figure 3. A notable increase began around 2013, followed by periodic dips in 2014 and 2020, both of which were followed by recoveries. Despite these downturns, there has been steady growth, with 2023 seeing the highest output of six articles. This upward trend reflects growing interest in the field, particularly regarding migration and self-identity, as interdisciplinary research in sociology, psychology, and cultural studies expands to explore the complexities of these topics.

**Country-wise production**

**Figure 4:**
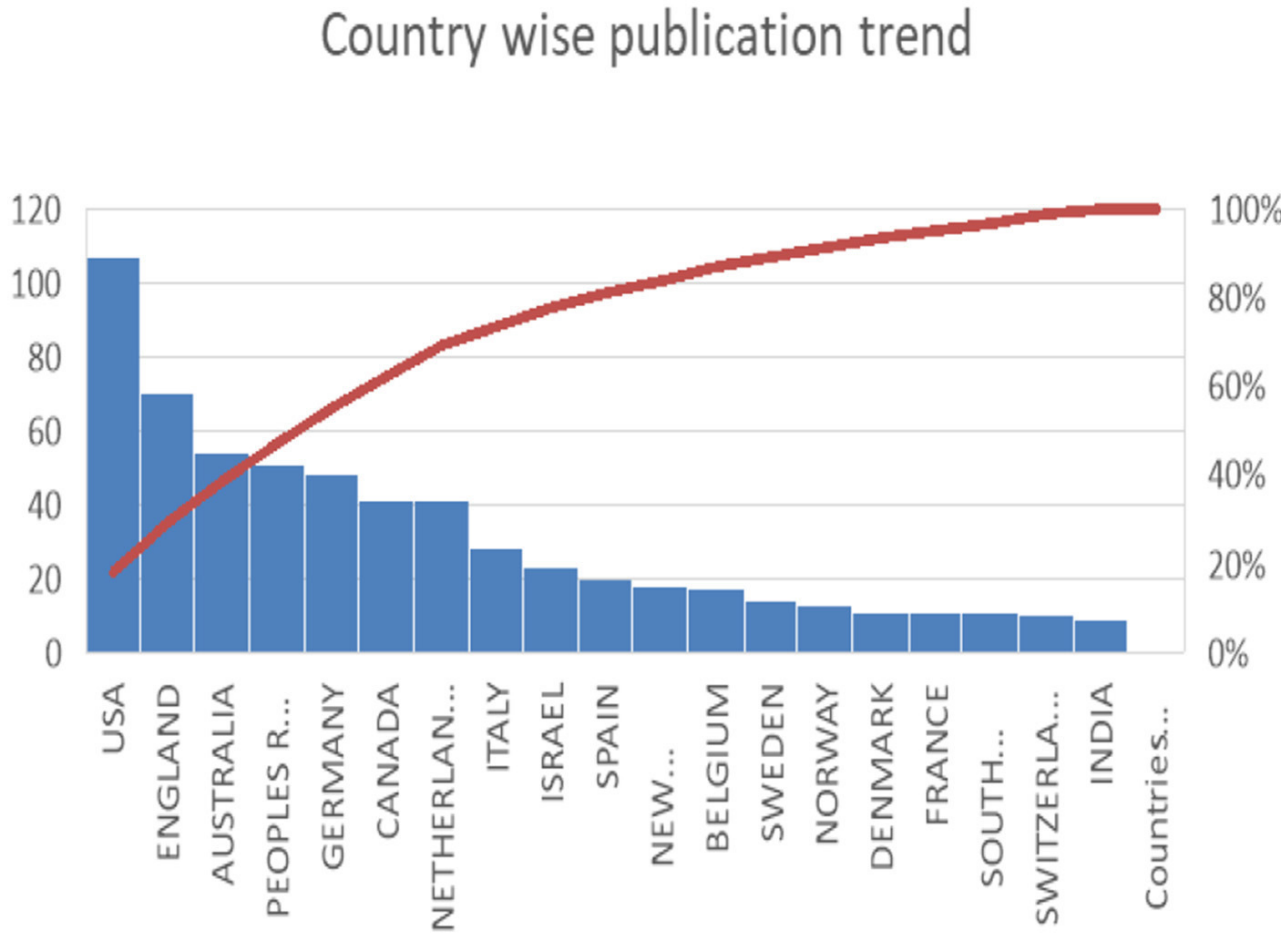
Country-wise publications trend depicting the number of publications per year.

**Figure 5:**
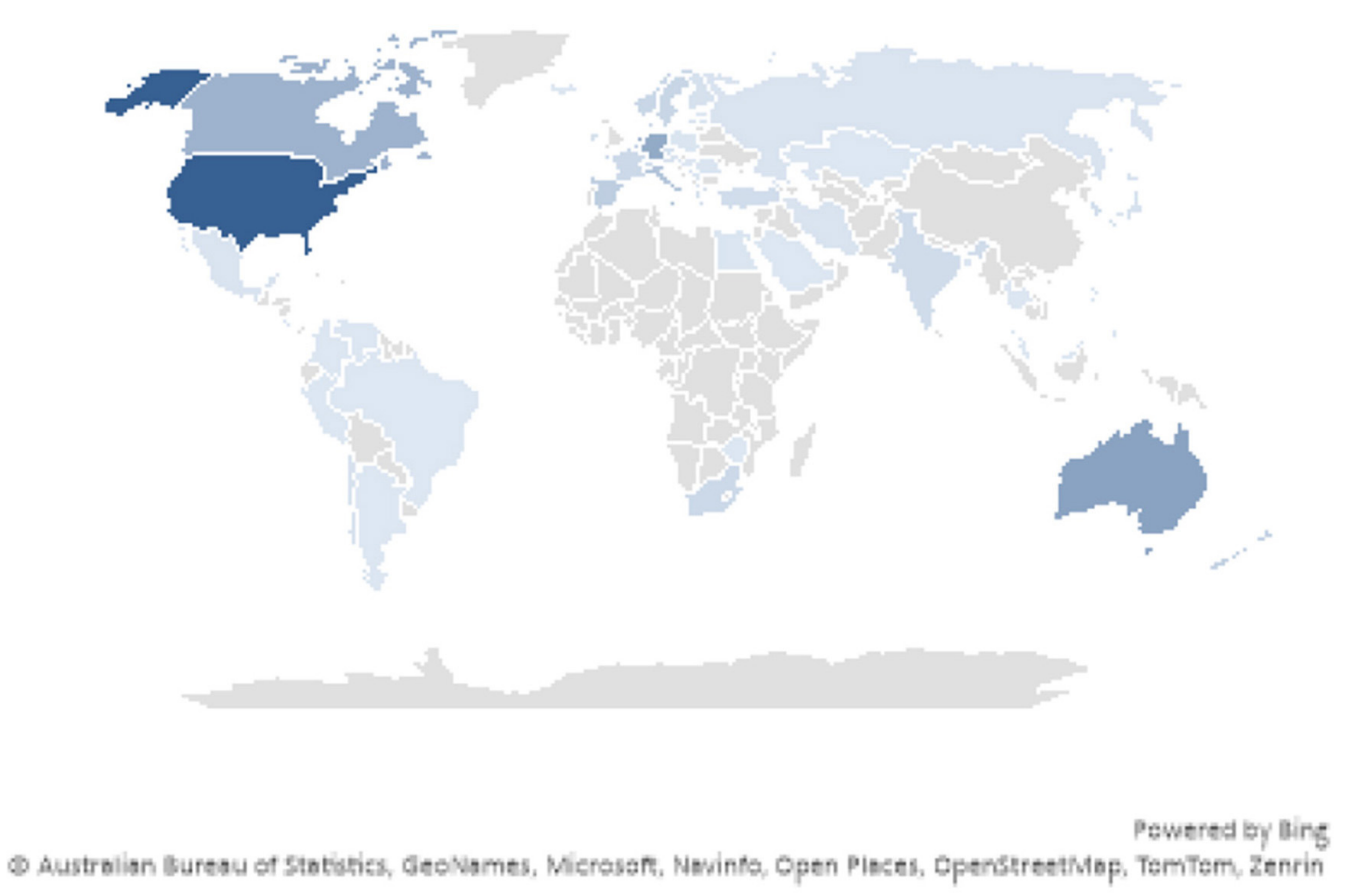
Country-wise publications trend depicting the number of publications per year.

Figures 4, 5 shows a country-wise publication trend, with the United States (USA) leading in the number of publications, followed by countries like England, Australia, and Germany. The figure highlights that the majority of research is concentrated in Western countries, particularly the USA and European nations. Eastern countries, such as India and China, are present but have significantly fewer publications on topics like self-identity and migration. This suggests that there is a gap in research from the Eastern context, indicating a lack of studies addressing the interplay between self-identity and migration in regions with different cultural, social, and economic dynamics.

**Co- citation network**

**Figure 6:**
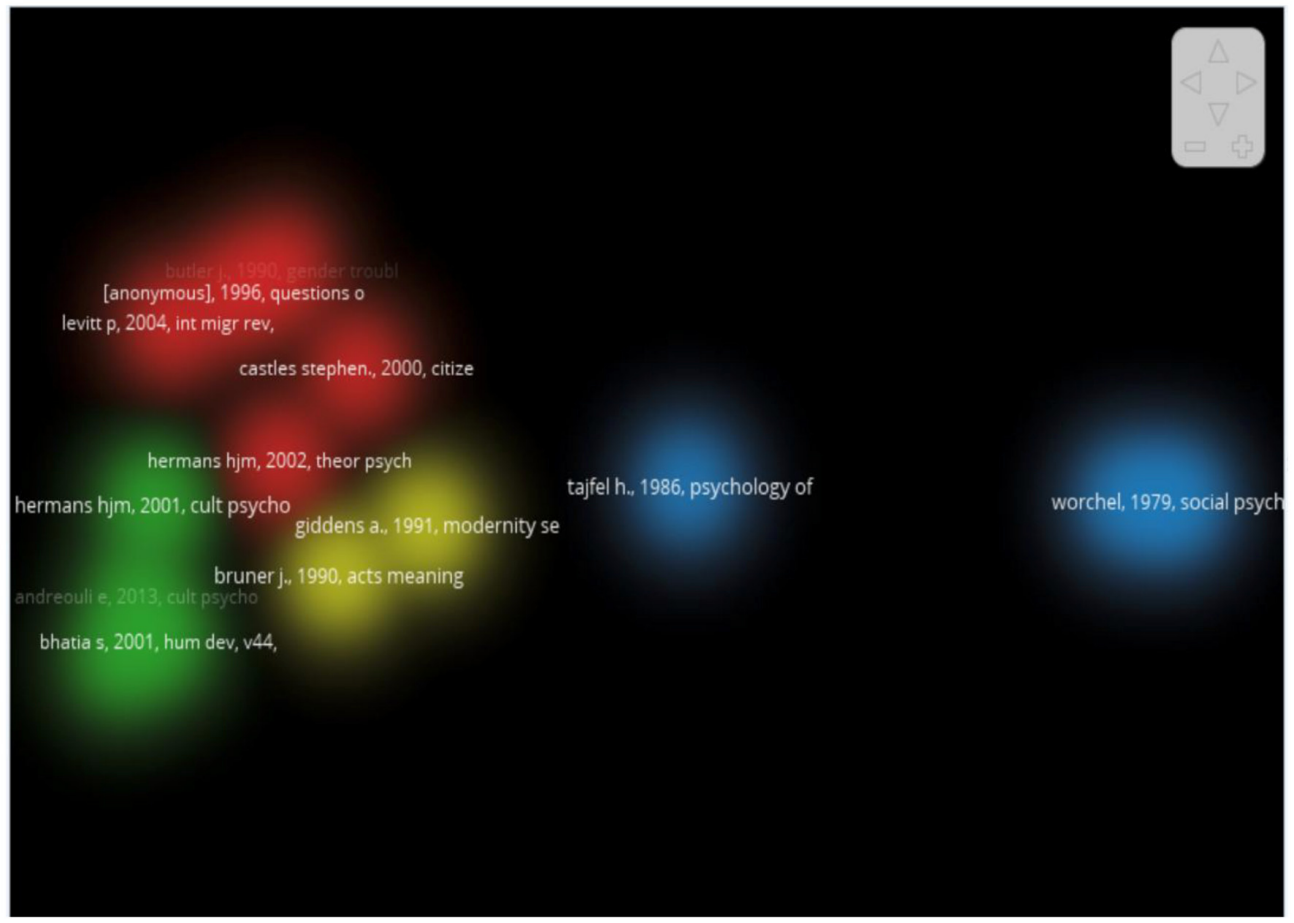
Document co-citation network for migration and self-identity related research.

This method assesses a publication's impact by counting the number of times it is cited, with citation count serving as a direct and objective measure of its influence. By analyzing citations, we can identify the most influential works within a research field. The Vosviewer map reveals clusters of references, each distinguished by color, signifying closely related groups of works that form the intellectual structure of the field as illustrated in Figure 6. The present study utilized the methodology proposed by Akbari (2021) to conduct a co-citation analysis of scholarly articles. The objective was to identify clusters of co-citations that pertain to the domains of self-identity and migration^.^

**ADO framework**

**Figure 7:**
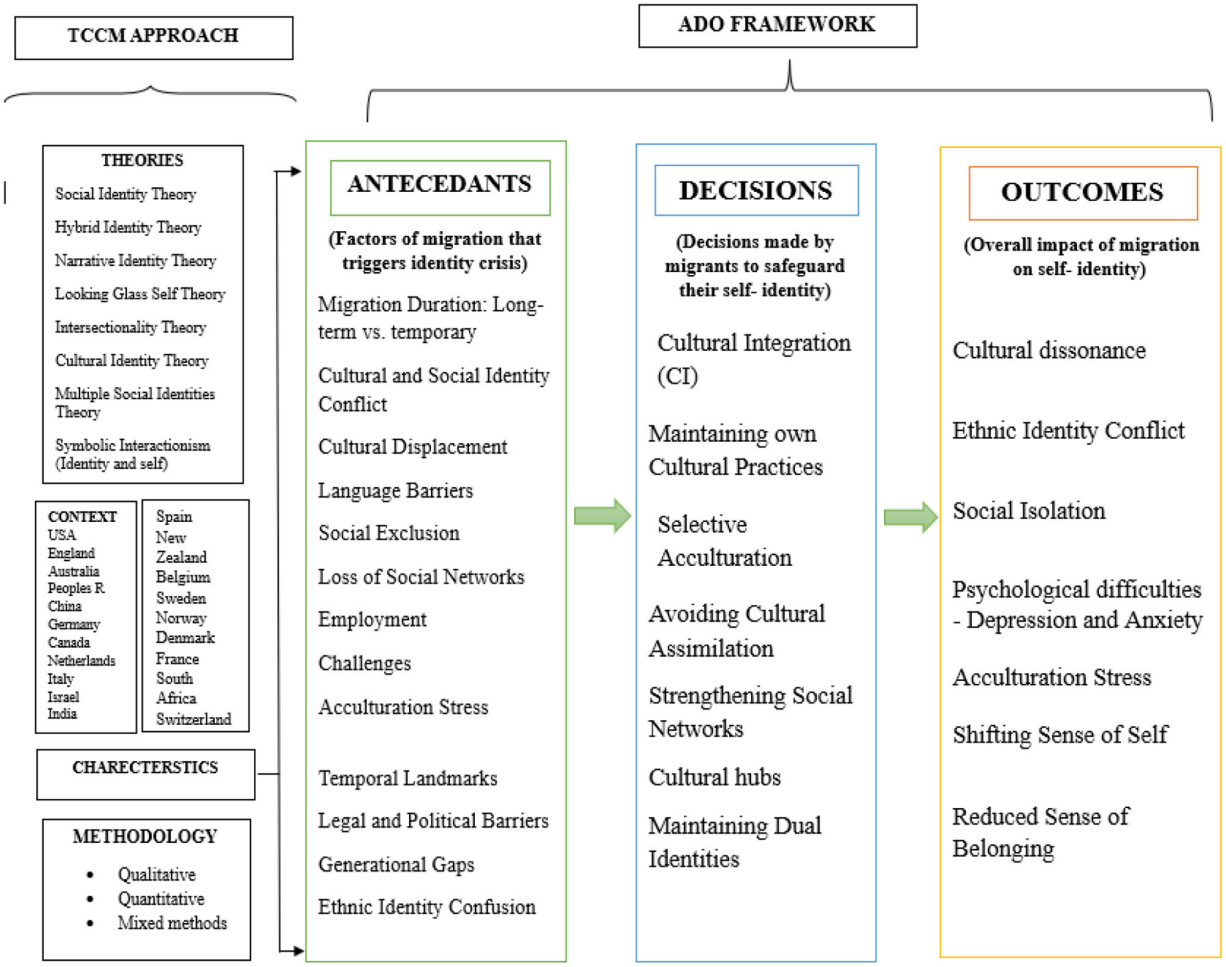
The TCCM-ADO framework for migration and self-identity related literature. Source: Author's own work.

The ADO framework (Paul and Benito, 2018) segments the review into three core components—antecedents, decisions, and outcomes (Table 1). Figure 7 demonstrates the consolidation of these components.

**Table 1:** Antecedents, decisions, and outcomes influencing Migration and Its Impact on Self-Identity.

	**Factors**	**References**
Antecedents (Factors of migration that trigger identity crisis)	Migration duration: Long-term vs. temporary	Eschbach and Gómez, 1998; Phinney, 2007
	Cultural and social identity conflict	Phinney, 2007; Jasinskaja-Lahti et al., 2011
	Cultural displacement	Marschall, 2017; Phinney, 2007
	Language barriers	Fillmore, 2020; Chen and Hong, 2016; Phinney, 2007
	Social exclusion	Chantah et al., 2024; Jiao and Qin, 2023
	Loss of social networks	Herman, 2004; Eschbach and Gómez, 1998; Bhugra, 2004
	Employment challenges	Cleton and Scuzzarello, 2024; Jiao and Qin, 2023
	Acculturation stress	Qian and Florence, 2023
	Temporal landmarks	Conway, 2023; Tartakovsky, 2009
	Legal and political barriers	Repke and Benet-Martínez, 2019; Bhugra, 2004
	Generational gaps	Phinney, 2007; Eschbach and Gómez, 1998; Herman, 2004
Decisions (Decisions made by migrants to safeguard their self-identity)	Cultural Integration (CI): Choose to assimilate or maintain cultural practices	Wang and Giovanis, 2024
	Maintaining own cultural practices	Cleton and Scuzzarello, 2024; Jiao and Qin, 2023
	Selective acculturation	Chantah et al., 2025
	Avoiding cultural assimilation	Chen and Hong, 2016
	Strengthening social networks	Repke and Benet-Martínez, 2019
	Maintaining dual identities	Conway, 2023
Outcome (Overall impact of migration on self-identity)	Cultural dissonance	
	Ethnic identity conflict	Khouri, 2016
	Social isolation	Chen and Hong, 2016
	Psychological difficulties - Depression and anxiety	Bhugra, 2004
	Acculturation stress	Tartakovsky, 2009
	Shifting sense of self	Marschall, 2017

Source: Author's own work.

**Antecedents: factors leading to migrant identity crisis**

Migration often disrupts an individual's sense of self, leading to identity struggles influenced by various factors. Migration duration plays a key role—long-term migrants often face deeper conflicts between their home and host cultures (Eschbach and Gómez, 1998; Phinney, 2007). This is intensified by cultural and social identity conflicts, where migrants feel torn between preserving their heritage and adapting to new norms (Phinney, 2007; Jasinskaja-Lahti et al., 2011).

Cultural displacement causes further disorientation, while language barriers isolate individuals, limiting communication and social integration (Marschall, 2017; Phinney, 2007). Social exclusion and loss of support networks add to feelings of alienation, while employment challenges and legal restrictions heighten insecurity (Chantah et al., 2025).

Additionally, acculturation stress, generational gaps, and ethnic identity confusion create internal conflicts, as migrants struggle to balance self-perception with societal expectations (Cleton and Scuzzarello, 2024; Repke and Benet-Martínez, 2019). These challenges collectively shape the identity crises many migrants face.

**Decisions: how migrants navigate identity**

To cope, migrants make conscious decisions about cultural integration. Some opt for full assimilation, adopting the host country's norms and letting go of their cultural roots to fit in more seamlessly (Qian and Florence, 2023). Others practice selective acculturation, choosing to retain key elements of their heritage while adapting selectively to the host culture, which allows them to preserve identity while navigating new settings (Chantah et al., 2025). Many migrants also focus on rebuilding social support by forming close-knit networks and creating cultural hubs—community spaces where shared language, traditions, and practices offer a sense of familiarity and belonging. Additionally, some develop a dual or bicultural identity, integrating aspects of both their heritage and host cultures into a cohesive self-concept, which helps them manage identity tensions and fosters flexible social integration (Repke and Benet-Martínez, 2019). These strategic choices not only influence personal wellbeing and identity coherence but also shape broader patterns of social inclusion and cohesion in multicultural societies.

**Outcomes: the impact of migration on identity**

The impact of migration on identity is deeply shaped by how migrants adapt to their new environment. For some, this process leads to cultural dissonance—a sense of confusion or discomfort when navigating conflicting cultural norms and expectations (Khouri, 2016). Many migrants experience ethnic identity conflict, feeling torn between loyalty to their heritage and the pressure to conform to the host culture. These internal struggles are often compounded by social isolation, especially when language barriers or discrimination limit connection with others (Chen and Hong, 2016).

Such conditions can contribute to psychological distress, including anxiety and depression (Keles, 2017; Bhugra, 2004). Acculturation stress, stemming from the challenges of cultural adaptation, further intensifies these emotional difficulties and can result in a shifting or fragmented sense of self (Tartakovsky, 2009; Marschall, 2017). Migrants may question their identity or feel disconnected from both cultures.

However, outcomes vary widely. While some struggle with identity loss, others manage to integrate both cultural worlds, forming a strong, bicultural identity that enhances resilience and belonging (Tartakovsky, 2009).

**iv. Methodology**

As identified in the past literature, a combination of research design methodologies has been utilized, including qualitative, quantitative, and mixed methods approaches. Data collection methodologies predominantly consist of surveys, questionnaires, semi-structured interviews, focus group discussions, and self-report measures. For data analysis, various techniques will be employed, such as narrative analysis, content analysis, thematic analysis, and to quantify relationships and test hypotheses, regression analysis and structural equation modeling have been used. An emerging trend has been seen in the use of phenomenological analysis. This diverse approach aims to explore multiple facets of the research question, contributing to a comprehensive understanding of the subject matter.

### 3.2 What are the dominant research topics in the study of migration's impact on self-identity?

**Figure 8:**
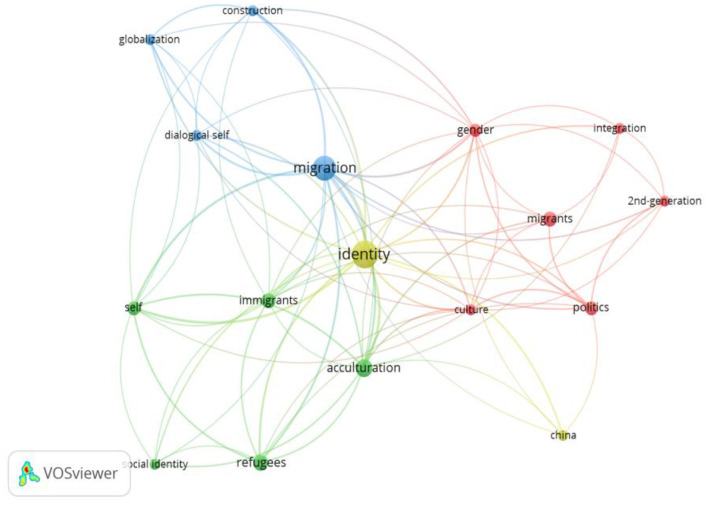
Keyword co-occurrence map.

This research question revolves around identifying the dominant research topics concerning the impact of migration on self-identity. This inquiry seeks to uncover the key areas and themes that have garnered significant scholarly attention within this field as illustrated in Figure 8. To address this question, we employ a keyword co-occurrence analysis.

#### 3.2.1 Key-word co-occurrence analysis

**Cluster 1: migration and globalization**

This cluster emphasizes the relationship between migration, globalization, and the construction of identity, focusing on how migratory processes affect the individual's sense of self and belonging. Migration is closely linked to globalization, as the interconnectedness of economies and cultures facilitates the movement of people across borders, which in turn shapes their identities in new ways. The concept of the dialogical self is crucial here, highlighting that individuals construct their identity through dialogues with their social environments, particularly as they move between different cultural contexts (Herman, 2004). This cluster explores how migrants must navigate and negotiate their identities in a rapidly changing global landscape, which may lead to hybrid identities, often formed through interaction with both the home and host cultures. Additionally, globalization influences how national boundaries are perceived and experienced, resulting in transnational identities that are less tied to specific geographic locations (Bauman, 2000). As people move across borders, they carry with them cultural norms, traditions, and beliefs, which can be challenged or reinforced in the global setting. Adding to this conversation, Gamsakhurdia's (2018, 2019) theory of proculturation offers a more flexible and realistic way to understand how migrants build their identities. Based on Dialogical Self Theory, this idea sees identity as something created through a back-and-forth exchange between different parts of a person's self. Migrants don't simply give up one culture and adopt another. Instead, they draw from many cultural influences—both old and new—and actively shape who they are through ongoing dialogue with these different cultural “voices” or “I-positions.” This approach sees identity as something fluid, sometimes messy or contradictory, and built through personal stories and experiences, rather than as a clear-cut process of fitting in.

**Cluster 2: gender, politics, and migration**

The gendered experience of migration and its intersection with politics is the focus of this cluster, particularly regarding how policies and societal norms influence the migration process. Gender plays a significant role in shaping the migration journey, as women and men often face different challenges related to labor market participation, family responsibilities, and legal status (Kofman, 2005). For example, migrant women may experience additional barriers, such as gender-based discrimination in employment or restricted access to social services, complicating their integration into the host society (Anthias, 2008). Political factors also shape the migrant experience, as state policies on immigration, integration, and citizenship directly affect their social and economic opportunities (Kofman, 2005). In this context, 2nd-generation immigrants—those born to migrant parents in the host country—are a particular focus, as they often navigate complex issues of integration and identity formation, balancing the cultural expectations of both their parents' homeland and their own (Portes et al., 2001). This generation frequently faces the challenge of forging a new, hybrid identity within a socio-political landscape that may either support or hinder their full acceptance.

**Cluster 3: the self, immigrants, and refugees**

This cluster examines the complex interplay between migration and identity formation, with a particular focus on the psychological and social identity challenges faced by immigrants and refugees in their host environments. Migration is not merely a geographical transition; it entails a profound reconfiguration of the self, as individuals must recalibrate their identity in response to new social, cultural, and economic realities (Berry, 1997). This process can be particularly disorienting and traumatic for refugees who are compelled to flee their homelands due to war, persecution, or environmental disasters (Zetter, 2007; Miller and Rasmussen, 2010).

A key dimension in this identity transformation is acculturative stress, which arises from the demands of adjusting to a new culture while maintaining one's heritage identity (Berry et al., 2006). This stress is amplified for those who experience discrimination, language barriers, and loss of social support systems (Schwartz et al., 2010). Refugees often endure “cultural bereavement,” a sense of grief and disconnection from their cultural roots, which may manifest in psychological distress, including depression and anxiety (Eisenbruch, 1991).

The social identity theory (Tajfel and Turner, 1979) is particularly relevant in understanding how migrants construct a sense of belonging in their new societies. According to this theory, people categorize themselves and others into in-groups (groups they belong to) and out-groups (groups they don't belong to). These group affiliations influence self-esteem and behaviors, particularly when individuals compare their in-group to out-groups to maintain a positive social identity. For migrants, the process of adjusting to a new society often involves navigating the tension between their heritage culture (the in-group) and the host culture (the out-group). As they interact with their new environment, migrants must balance the desire to preserve their cultural identity with the pressures to integrate into the host society, which may be marked by differing social norms and values. Social Identity Theory helps explain how this negotiation of identities can influence a migrant's sense of belonging. Those who can maintain strong ties to their heritage culture while also engaging with the host society often report higher levels of bicultural identity and wellbeing. On the other hand, migrants who experience marginalization or exclusion from the host society may face identity conflict and lower levels of psychological adjustment (Benet-Martínez and Haritatos, 2005). The host society's reception, ranging from inclusive multiculturalism to exclusionary nationalism, plays a significant role in shaping migrant identity (Esses et al., 2013).

Moreover, labeling and legal classification—particularly for refugees—can lead to a “labeling paradox,” wherein individuals are simultaneously provided protection and subjected to social exclusion and stigma (Zetter, 2007). The bureaucratic and media-driven labels such as “illegal,” “asylum seeker,” or “economic migrant” affect how migrants perceive themselves and how they are treated by institutions and host communities (Crawley and Skleparis, 2018). These imposed identities often conflict with migrants' self-concepts and contribute to a fragmented or hybrid identity.

**Cluster 4: identity and acculturation**

This cluster revolves around the themes of identity, acculturation, and the negotiation of cultural integration for migrants in new societies. The process of acculturation refers to how migrants adapt to and adopt the cultural practices of their host country while simultaneously maintaining their cultural heritage (Berry, 1997). This balancing act between maintaining one's original identity and adapting to a new culture can result in a hybrid or bicultural identity, where elements from both the home and host cultures coexist (Phinney, 1990). Theories of acculturation emphasize that migrants must navigate complex cultural landscapes, where pressures to assimilate can conflict with the desire to preserve one's cultural traditions (Berry et al., 2006). The cultural identity of migrants is fluid and often evolves as they engage with the dominant culture of the host society, leading to varying degrees of integration, assimilation, or separation depending on individual experiences and societal attitudes (Berry, 1997). Scholars in this area often explore the psychological impacts of acculturation, noting that successful adaptation is often linked to the ability to maintain a positive sense of one's cultural identity while engaging with the new culture (Phinney, 1990).

### 3.3 RQ-3: what are the primary themes or areas in the study of migration's impact on self-identity, as identified through thematic mapping of current literature?

**Figure 9:**
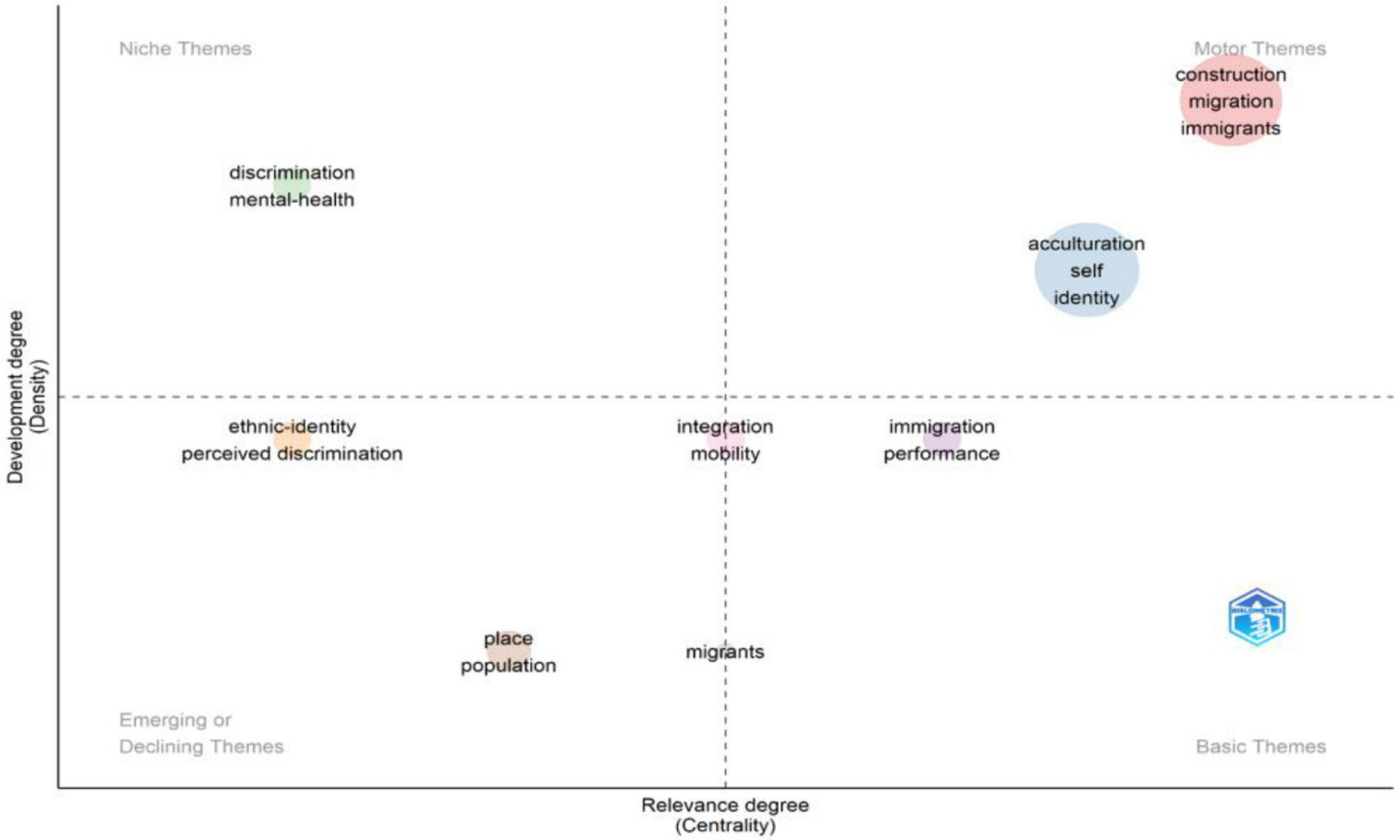
Thematic mapping of clusters.

**Figure 10:**
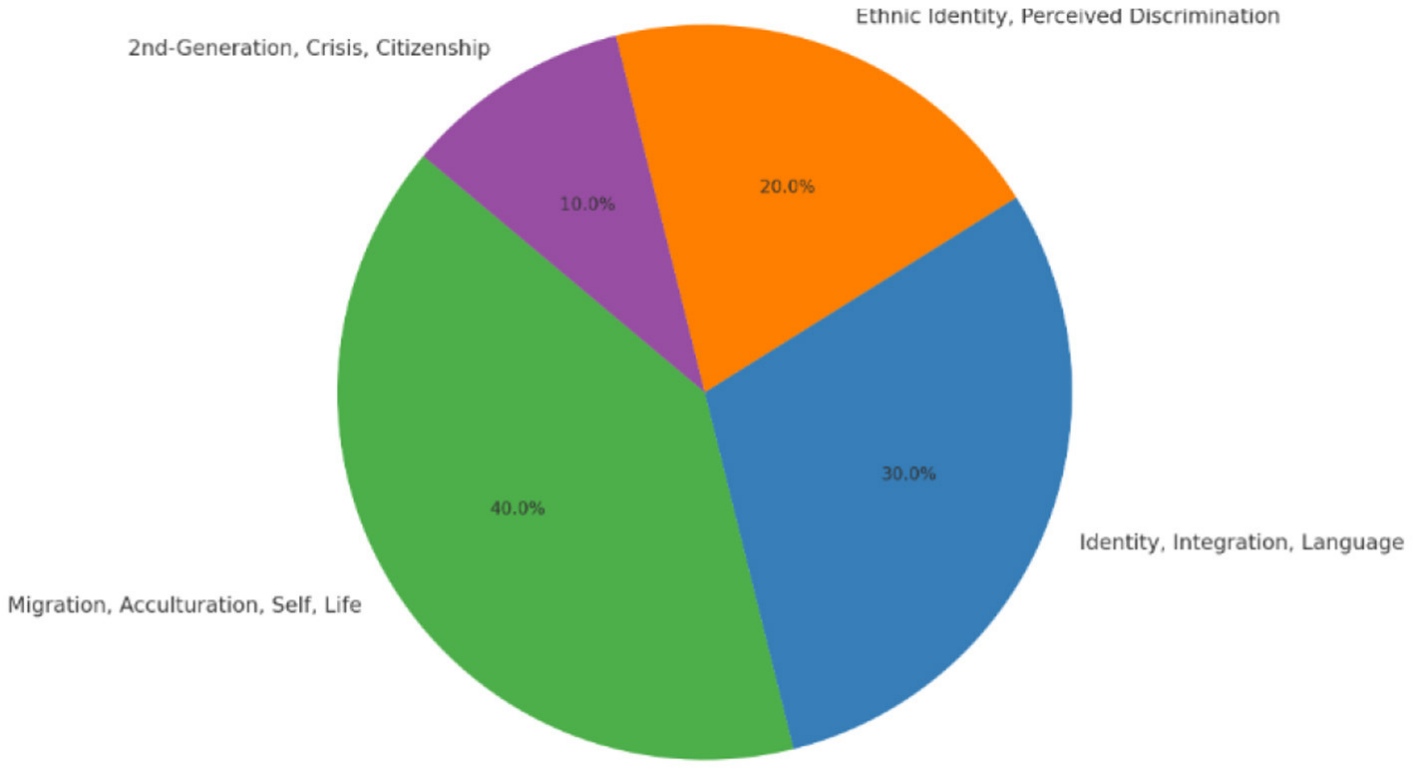
Distribution of articles across thematic clusters.

Finally, the third research question seeks to identify potential future research avenues that could address current gaps in the literature and suggest new perspectives on how to better nurture the self-identity of migrants through thematic mapping (Figure 9). Figure 10 depicts the distribution of articles across the thematic clusters.

#### 3.3.1 Thematic mapping

Cobo et al. (2011) categorize thematic maps into four quadrants based on how strongly themes are linked to each other and their prominence in the broader field. A conceptual thematic map involves clustering related keywords into subgroups that represent focal points or significant research issues drawing substantial scholarly interest and resources. Centrality measures how well a theme connects to other themes. If a theme is highly connected, it means it's important and interacts with many other topics. Density shows how well-connected the keywords are within a theme. High density means that the terms related to a theme are strongly linked, indicating that the theme is well-developed.

The findings reveal a diverse yet interconnected set of thematic areas that define how migration influences the formation, disruption, and reconstruction of self-identity. Central to this field are themes such as migration, acculturation, self-construction, and life transitions, which are considered well-established and foundational in migration studies. These themes act as motor themes (quadrant 1), driving and shaping the research landscape by offering core insights that connect multiple areas of inquiry. For example, Berry's (1997) acculturation framework, comprising integration, assimilation, separation, and marginalization, provides a robust lens for understanding migrants' navigation between host and heritage cultures. Research suggests that bicultural or integrated strategies generally result in better psychological outcomes and stronger identity coherence (Nguyen and Benet-Martínez, 2013).

Quadrant 2 represents basic themes—broad and foundational topics that are central to the field but less developed in terms of research density. These themes have high relevance (centrality) across multiple areas of study and serve as essential building blocks for understanding the migration experience. In migration and identity studies, key themes in this quadrant include identity, integration, language, and the performative dimensions of migration. Identity is a central concern, as it shapes how migrants perceive themselves and how they are perceived by others. It is influenced by both their ethnic or cultural background and their lived experiences in the host society (Phinney et al., 2001). Identity is not static—it is continually negotiated and reconstructed through everyday interactions and social contexts. Integration goes beyond physical relocation; it involves becoming embedded in the social, economic, cultural, and political life of the host society. As Berry (1997) argues, successful integration contributes to both individual wellbeing and societal cohesion. It encompasses mutual adaptation and participation, requiring effort from both migrants and the host communities. Language plays a pivotal role in both identity and integration. It serves as a tool for communication, belonging, and cultural expression, and often becomes a marker of inclusion or exclusion. Proficiency in the host country's language can significantly impact access to opportunities and the ability to engage fully in society. Importantly, these themes intersect in the performance of migration—how migrants enact, express, and negotiate their identities and belonging in both everyday life and cultural contexts. Performance here is not limited to artistic expression but includes the symbolic and practical ways migrants adapt, assert, and integrate themselves through language use, cultural practices, public presence, and social participation.

In addition to these core themes, perceived discrimination and mental health, function as niche themes. Niche themes in Quadrant 3 are well-developed but have limited influence outside their specific research areas. Examples include Ethnic Identity and Perceived Discrimination, which are central to migration and identity studies but remain specialized topics. Ethnic Identity explores how migrants maintain connections to their heritage while adapting to a new culture, shaped by both societal pressures and self-identification (Phinney, 1990). Perceived Discrimination, closely linked to ethnic identity, examines how prejudice and stereotyping impact migrants' mental health, increasing stress and identity conflicts (Pascoe and Richman, 2009). These themes are deeply relevant to migrant experiences but remain somewhat isolated in broader migration research due to their focus on specific groups.

Emerging themes in Quadrant 4 are still developing and offer new research opportunities. Topics like Second-Generation Identity, Crisis, and Citizenship are gaining attention, particularly in understanding the identity struggles of migrants' children. Second-generation immigrants, born in host countries to migrant parents, often face dual cultural identities and must navigate integration challenges (Portes et al., 2001). Crisis relates to identity struggles caused by marginalization, while Citizenship explores how migrants gain legal rights and societal recognition, shaping their sense of belonging (Bloemraad, 2007). These themes, though currently underexplored, hold potential for future research as second-generation populations grow. Additionally, intersectionality—the interplay of race, ethnicity, gender, and religion—further shapes migrant experiences, as individuals from marginalized backgrounds may face compounded discrimination, making identity adaptation more complex (Cleton and Scuzzarello, 2024). For instance, A Black Muslim woman migrating to a Western country may not only navigate cultural adjustments but also encounter biases based on race, gender, and faith. Meanwhile, certain themes, such as assimilation (in its traditional unidirectional sense) and cultural loss, can be considered declining themes, as scholarship increasingly critiques these models for oversimplifying the complexities of migrant identity negotiation and favors more dynamic frameworks like integration and biculturalism (Alba and Nee, 2003).

## 4 Conclusion

This study offers a critical bibliometric analysis of the relationship between migration and self-identity, shedding light on key themes, emerging trends, and areas that require further exploration. Through the identification of four primary clusters, acculturation and identity formation, migration and globalization, gender and migration, and the psychological experiences of immigrants and refugees, the analysis underscores the multifaceted nature of migration's impact on self-identity and mental wellbeing.

One of the most prominent themes identified was acculturation, particularly the challenges and strategies migrants employ to balance their heritage culture with that of the host society. Berry's (1997) framework on acculturation continues to be a foundational theory in understanding this process. Additionally, the concept of bicultural identity, as outlined by Phinney et al. (2001), remains critical in assessing how migrants negotiate multiple cultural identities. These theoretical perspectives underscore the importance of cultural integration, which has profound implications for both individual wellbeing and the broader societal dynamics in multicultural settings.

The study's contributions are particularly timely and relevant in the present socio-political context, where migration continues to be a central issue across the globe. As migration patterns are shaped by various factors such as conflict, climate change, and economic instability, understanding the psychological experiences of migrants has never been more urgent. In the wake of the ongoing refugee crisis and the rise of nationalist movements in many host countries, the need for inclusive policies that support both the psychological and social wellbeing of migrants has become paramount. The insights gained from this study can help inform these policies, particularly in fostering environments where migrants can successfully integrate without losing their cultural identities.

Moreover, the findings of this study have direct implications for the development of culturally sensitive mental health programs. The growing emphasis on multiculturalism and diversity across the world necessitates the creation of interventions that not only address migrant mental health but also respect and incorporate their cultural backgrounds. This aligns with the broader movement in psychology and social sciences toward a more intersectional and holistic understanding of identity that goes beyond simplistic dichotomies of “us” vs. “them.”

However, despite these significant contributions, several important gaps persist. Notably, there is a lack of research exploring the experiences of second-generation immigrants. This subgroup is crucial for understanding how the children of migrants navigate their identities in a hybrid cultural environment. Additionally, there is a notable scarcity of intervention-based research aimed at improving the mental health of migrants. Existing studies have largely focused on descriptive analyses rather than applied interventions that can directly benefit migrant populations.

In light of these gaps, future research must focus on resilience-building strategies for migrant populations, particularly in areas that address mental health, community engagement, and the development of bicultural competencies. Longitudinal studies examining the long-term psychological outcomes of migration would provide valuable insights into how the migrant experience unfolds over time. Furthermore, interventions that take into account the intersectionality of identity, such as gender, socioeconomic status, and ethnic background, should be prioritized to create more nuanced and effective programs.

Finally, the study's findings emphasize the importance of integrating migration research into broader discussions on social justice and policy reform. By building on existing theoretical frameworks and incorporating the voices of migrants themselves, researchers and policymakers can create environments that foster both integration and belonging. As migration continues to shape the social fabric of societies worldwide, this study contributes to the growing need for a more empathetic and evidence-based approach to migrant wellbeing, ensuring that both migrants and the host community benefit from the process of migration.

In conclusion, this study not only fills critical gaps in the migration literature but also offers recommendations that can shape both future research and policy. As migration increasingly impacts societies globally, understanding how it influences self-identity and mental health is essential for creating a more inclusive and supportive world for migrants. By integrating the components of Social Identity Theory into migration research, scholars can better capture the dynamic interplay between personal identity, group membership, and sociopolitical context. Addressing the identified gaps will allow future research to make significant strides in improving the resilience, integration, and wellbeing of migrant populations.

## 5 Implications

### 5.1 Managerial implications

For managers, recognizing how migration shapes self-identity is essential for creating an inclusive, supportive, and high-performing workplace. Providing resources like mentorship programs and counseling services can help employees navigate identity conflicts, boosting their confidence, job satisfaction, and psychological safety.

Effective leadership requires flexibility, acknowledging that identity is fluid, and empowering employees to bring their authentic selves to work. Organizations should implement cultural sensitivity training to foster collaboration across diverse backgrounds, helping to reduce biases.

A strong diversity and inclusion strategy should celebrate hybrid identities, valuing the unique perspectives that employees from different cultural backgrounds bring. Embracing this diversity not only enhances workplace culture but also drives innovation, problem-solving, and creativity, making inclusion a strategic advantage for organizations.

Managers can also leverage the diverse perspectives of migrant employees to enhance creativity and global market understanding. Using insights from migrant identities can be beneficial to develop culturally inclusive products and services, broadening the organization's customer base.

### 5.2 Theoretical implications

The migration experience offers deep insights into self-identity (Table 2), highlighting the need for more flexible and inclusive theoretical frameworks. Traditional models often assume a fixed sense of identity, but migrants frequently develop hybrid identities, navigating between their cultural heritage and the expectations of their new environment. To fully capture this complexity, future theories must account for identity conflicts, adaptability, and the fluid nature of self-concept.

**Table 2:** Dimensions of migration and their impact on self-identity.

**Dimension of migration**	**Impact on self-identity**
Cultural dimension	- Exposure to new traditions can expand or challenge pre-existing cultural identities.
	- Individuals may experience “cultural hybridity,” blending aspects of both home and host cultures.
	- Changes in social networks (friends, family, and community) can disrupt social identity, leading to feelings of isolation or a search for new connections.
Social dimension	- Migrants may redefine their role within new societal structures, reshaping their sense of self.
	- Discrimination or acceptance in the host society can affect self-esteem and perceived self-worth.
	- Employment opportunities or financial struggles can impact self-perception, especially if the individual experiences downward mobility or deskilling.
Economic dimension	- Success in economic integration can enhance confidence and reshape self-identity in a more positive light.
	- Migrants may experience identity crises due to conflicting loyalties between home and host cultures (bicultural identity).
Psychological dimension	- Emotional challenges, such as homesickness or trauma, can affect self-perception and personal growth.
	- Displacement or relocation may lead to a loss of connection to physical places tied to identity (e.g., hometowns, landscapes, or sacred spaces).
Geographical dimension	- Settling in new environments might require developing a new sense of place and belonging.
	- Migrants may face shifts in civic identity, such as acquiring new citizenship or feeling excluded from political processes in the host country.
Political dimension	- Political marginalization can lead to activism and reassertion of identity tied to origin.
	- Learning a new language may reshape self-expression and identity.
Linguistic dimension	- Language loss or retention becomes a marker of cultural preservation or assimilation.
	- Communication barriers can lead to feelings of invisibility or disconnection from the host community.
	- Migrants may adapt religious practices or reassert spiritual identities in response to differing beliefs in the host country.
Religious/Spiritual dimension	- Religious institutions in diaspora communities often become central to maintaining identity and fostering a sense of belonging.
	- First-generation migrants may cling strongly to their original identity, while second-generation migrants often navigate a dual identity.
Generational dimension	- Intergenerational conflict can emerge over differing levels of assimilation or cultural retention.
	- Migration may challenge or reinforce gender roles, depending on the cultural context of the host society.
Gender dimension	- Women and LGBTQ+ migrants may face unique identity challenges tied to societal expectations and discrimination.

Source: Author's own work.

Theories on cultural hybridization should also explore how blending perspectives fosters cross-cultural understanding and innovation, particularly in workplaces that thrive on diversity. Integrating identity fluidity into social identity theory can offer a more nuanced and realistic perspective on how individuals redefine themselves in today's globalized world.

Furthermore, acculturation models (Berry, 1997) often present adaptation as a linear process, categorizing migrants into assimilation, integration, separation, or marginalization. However, real-world experiences are far more nonlinear and dynamic. Future frameworks should reframe acculturation as a circular, multidirectional process.

## 6 Future research agenda

This review highlights several gaps in migration and self-identity research that need further exploration. While the psychological effects of migration are well-documented, there is a lack of intervention-based studies focused on preventive mental health strategies. Most existing interventions primarily address diagnosed conditions, overlooking the needs of at-risk migrant groups who could benefit from mental health programs, community support, and cultural preservation initiatives (Purgato et al., 2021).

Another key gap is the Western-centric focus of much of the literature, leaving migration experiences in Asia, Africa, and Latin America underexplored. Understanding regional differences in identity negotiation and adaptation would offer a more comprehensive, global perspective. Comparative studies could also shed light on how cultural contexts shape acculturation experiences.

Additionally, second-generation immigrants require more attention, as they often struggle with dual identities, balancing their heritage with the norms of their host society. Their experiences with belonging, integration, and identity formation differ significantly from those of first-generation migrants and warrant further study (Portes et al., 2001).

Future research should also explore the cognitive and emotional processes that shape identity during migration. Longitudinal studies tracking migrants over time could provide insights into how self-identity evolves in response to life transitions. With the growing role of social media and digital communities, it is also essential to examine how online spaces influence migrant identity and belonging. By addressing these gaps, future research can contribute to practical strategies that enhance migrant wellbeing, integration, and inclusion, ultimately fostering more diverse and welcoming societies.

## 7 Limitations of the study

While this study provides a robust and structured examination of the interplay between migration and self-identity through the TCCM framework and bibliometric analysis, several limitations should be noted to frame the scope of its contributions. The exclusive reliance on the Web of Science database, although ensuring access to high-quality and peer-reviewed literature, may have inadvertently excluded relevant studies available in other databases or gray literature, potentially limiting the comprehensiveness of the review. Additionally, the bibliometric methodology, while effective in mapping research trends and thematic developments, inherently emphasizes quantitative patterns over the nuanced, lived experiences that qualitative methods, such as ethnography or in-depth interviews, can reveal. Another limitation lies in the level of abstraction in bibliometric clustering and thematic mapping; while these tools are effective for synthesizing large volumes of data, they may oversimplify complex and overlapping concepts. These limitations do not undermine the value of the current analysis but rather highlight opportunities for future studies to expand and deepen the understanding of migrant identity by incorporating more diverse data sources, languages, and methodological approaches.

## Data Availability

The original contributions presented in the study are included in the article/supplementary material, further inquiries can be directed to the corresponding author.
